# The associations between work-related factors and temporomandibular disorders among female full-time employees: findings from the Fourth Korea National Health and Nutrition Examination Survey IV (2007–2009)

**DOI:** 10.1186/s40557-018-0253-9

**Published:** 2018-06-20

**Authors:** Wook Han, Soon-Chan Kwon, Yong-Jin Lee, Chan Park, Eun-Chul Jang

**Affiliations:** 1 Department of Occupational and Environmental Medicine, Soonchunhyang University Cheonan Hospital, 31, Suncheonhyang 6-gil, Dongnam-gu, Cheonan-si, Chungcheongnam-do 31151 Republic of Korea; 2 Environmental Health Center for Asbestos, Soonchunhyang University Cheonan Hospital, 67, Suncheonhyang 3-gil, Dongnam-gu, Cheonan-si, Chungcheongnam-do 31151 Republic of Korea

**Keywords:** Working hours, Temporomandibular disorders (TMD)

## Abstract

**Background:**

The aim of this study was to investigate the association between work-related factors and temporomandibular disorders (TMD) among female full-time employees using representative data from a national population-based survey.

**Methods:**

Data from the Fourth Korea National Health and Nutrition Examination Survey IV (2007–2009) were used to analyze 1,612 women. Complex samples logistic regression was applied for adjusting for general characteristics and work-related factors to examine the association between work-related factors and TMD.

**Results:**

The prevalence of TMD was 12.8% in this study population. With respect to age, educational status, marital status, problem drinking, exercise, and stress, there were statistically significant differences in the prevalence of TMD. In logistic regression analyses on complex samples, based on 40 h or less per week, odds ratios (ORs) for respondents who worked 40–48 working hours, 49–60 working hours, and more than 60 h were 1.16 (95% confidence interval (CI) 0.69–1.94), 1.41 (95% CI 0.79–2.54), and 2.43 (95% CI 1.29–4.59), after adjusting for general characteristics, working schedule, employment status, and occupation.

**Conclusions:**

This study found that long working hours were significantly associated with TMD in Korean female full-time employees.

## Background

Companies are introducing innovative technologies in an effort to enhance production and efficiency in today’s competitive global market. The accompanying changes in the occupational environment such as longer working hours, more temporary workers, and greater job instability have resulted in a greater workload, faster pace of work, and greater complexity of work for many workers; accordingly, their physical and psychological burden has increased [1].

Long working hours are reported to be associated with all-cause mortality and have a negative effect on circulatory system diseases, diabetes, depression, anxiety, and other types of mental disorders as well as sleep patterns, cognitive function, and health-related behaviors [2–4]. Furthermore, temporary workers are more likely to experience psychological distress and depression than regular workers [5, 6].

Temporomandibular disorders (TMD) are one of the main contributors to pain of the face and are the second most common musculoskeletal condition after chronic low back pain [7]. TMD is a term that refers to all types of pain and functional problems in the masticatory muscles and jaw joints [8]. Some of the most common features of TMD include pain in the face and in front of the ear, limited jaw movement, and clicking or popping noises in the joint when the jaw moves [9].

Women show a higher level of TMD prevalence (2 to 4 times) than men [9]. Sex hormones, especially estrogens, play an important role in the pathogenesis of masticatory muscles and increase sensitivity to pain in TMD [10, 11]. Furthermore, many research articles have reported that women are considered to be more typically associated with TMD and pain control for TMD, because women are more sensitive to the development of pain [11, 12]. In addition to physiological factors, psychosocial factors such as depression, stress, and anxiety are correlated with TMD, and such factors occur more frequently among women than men [13, 14]. However, studies among women on the association between occupational environment and TMD are quite limited in Korea as well as in other countries.

Therefore, this research aimed to identify the association between TMD and work-related factors, such as long working hours and type of employment among female full-time workers in the Fourth Korea National Health and Nutrition Examination Survey (KNHANES IV).

## Subjects and method

### Study population

The KNHANES is a nationally representative health and nutrition examination survey in Korea conducted to establish and evaluate policy goals and provide policy references for a national health promotion program. The first survey of the Fourth KNHANES was conducted from July to December 2007, the second and third surveys of the Fourth KNHANES were performed from January to December in 2008 and 2009.

The Fourth KNHANES introduced rolling survey sampling in each of the 3 years of the survey, with probability samples representing the Korean population; rolling samples were independent from and homogenous with each other. In addition, three-stage stratified cluster sampling was used in the Fourth KNHANES. The first sampling was conducted based on *dongs* (neighborhood), *eups* (towns), and *myeons* (townships) in 11 cities and provinces of Korea. Participants were stratified and categorized into 29 strata based on age groups and percentages in *dongs*, *eups*, and *myeons*. The second sampling was based on *gus* (districts) whereas the third sampling was performed among households.

The health questionnaire and examination were conducted in a mobile medical examination center. The questionnaire was administered as an interview or was self-administered depending on the question items; examinations were performed by direct measurement, observation, and sample analysis.

There were 24,871 participants in the Fourth KNHANES; the percentage of those participating in all survey years was 78.4%. In this study, we analyzed the information of 1,612 full-time female workers, aged between 20 and 64 years, who answered the questionnaire. All participants provided written informed consent, and the institutional review board (IRB) of the Korea Centers for Disease Control and Prevention (KCDC) approved the study (IRB: 2007–02-CON-04-P, 2008-04EXP-01-C, 2009-01CON-03-2C) [15].

### General characteristics of study population

Age was categorized into three groups (20–39, 40–54, and 55–64 years). Respondents were also divided into four income groups (low, mid-low, mid-high, and high) and four groups by educational attainment (elementary school, middle school, high school, and college or higher). Marital status included single and married participant groups. Among respondents who drank alcohol in a given year, women were considered problem drinkers if they consumed five glasses or more of alcohol per occasion and drank twice or more every week. Smoking status was divided into three groups (current smoker, ex-smoker, and nonsmoker). For exercise, we categorized respondents according to whether they had engaged in vigorous-intensity physical activity for 20 min or longer per occasion and 3 days or more per week in the previous week (vigorous intensity was defined as feeling more exhausted or breathing more rapidly than usual). Respondents were also grouped based on whether they slept 6 hours and more or fewer than 6 hours a day. To evaluate obesity, we categorized respondents into three groups based on body mass index (BMI): < 18.5 kg/m^2^ (underweight), 18.5–24.9 kg/m^2^ (normal), and ≥ 25 kg/m^2^ (overweight). Two participant groups were formed according to whether they currently had osteoarthritis or rheumatoid arthritis (yes or no). In addition, respondents were grouped as those who perceived little stress and those who perceived a lot of stress. Self-rated health was surveyed on a five-point scale and respondents were classified as follows: ratings of “very good” and “good” were defined as “healthy”; other ratings were regarded as “not healthy”. Finally, respondents were queried whether they had depression in the previous 2 weeks in a row during the previous year (yes or no).

### Work-related factors

We grouped respondents by working hours per week: < 40 h, 40–48 h, 49–60 h, and > 60 h. The categories were based on 48 h of work, the maximum working hours set forth by the European Union (EU) [16], and 60 h, working hours recognized as a possible cause of death by overwork in Korea [17]. Regarding working schedule, respondents were classified as day workers if they worked between 6 a.m. and 6 p.m. and shift workers for all other work schedules. The type of employment was grouped into regular and temporary workers. Workers skilled in agriculture, forestry, and fishery; technicians; equipment, machine, and assembly workers; and low-skill workers were considered manual workers whereas all other occupations were categorized as non-manual workers.

### The definition of TMD

The questions pertaining to dental examinations in the Fourth KNHANES were questions suggested by the American Academy of Orofacial Pain (AAOP). AAOP questions show good reliability and validity for TMD screening according to Research Diagnostic Criteria for Temporomandibular Disorders (RDC/TMD) [18].

Dentists ask the survey participants the following questions in person and objectively examine the current status [19]:

1. a clicking sound in both or one of your jaws near the ear when you open your mouth
2. a contraction or pain inside your ear or around your temples or cheeks
3. pain or discomfort when you open your mouth, difficulty opening your mouth, or a dislocated jaw

When the participants had any of these three, they were defined as having TMD.

### Statistical analysis

Because the Fourth KNHANES used a complex sample design, we applied survey modules and weights in this study. A descriptive analysis was performed of survey participants’ demographic and behavioral characteristics and work-related factors. To identify the factors associated with TMD, chi-squared tests were conducted on complex samples. To estimate the odds ratios (ORs) and 95% confidence intervals (CIs), we adjusted for general characteristics and work-related factors, and then performed a logistic regression analysis on complex samples. All statistical analyses were performed using SPSS Version 19.0 (SPSS Inc., Chicago, IL, USA), and *p*-values less than 0.05 were considered to indicate statistical significance.

## Results

### General characteristics

Among the 1,612 female respondents, there were 207 (12.8%) with TMD. With respect to age, educational status, marital status, problem drinking, exercise, and stress, there were statistically significant differences in the prevalence of TMD (Table 1). However, household income, smoking, sleep duration, rheumatoid arthritis, osteoarthritis, self-rated health, and depressive symptoms showed no significant differences. For age, respondents aged 20–39 years showed the highest prevalence of TMD (18.4%). With respect to education, women with college-level education and higher had the highest prevalence of TMD (17.4%). For marital status, single people had a higher prevalence of TMD (21.1%) than married ones (9.6%). Regarding alcohol use, TMD prevalence among the problem drinker group (25.1%) was higher than the non-problem drinker group (12.2%). Women who exercised showed a higher prevalence of TMD (18.3%) than those who did not (12.3%), and respondents who felt a lot of stress had higher prevalence of TMD (18.9%) than those who felt little stress (9.9%).

**Table 1:** The prevalence of temporomandibular disorders (TMD) according to general characteristics of study population

	Prevalence of TMD		
	No N (%)	Yes N(%)	*P* value^a^
Total	1,405(87.2)	207(12.8)	
Age (years)			
20–39	647(81.6)	144(18.4)	< 0.001
40–54	579(92.7)	47(7.3)	
55–64	179(94.1)	16(5.9)	
Household income			
1Q(High)	485(85.4)	79(14.6)	0.526
2Q	426(88.0)	58(12.0)	
3Q	360(88.4)	46(11.6)	
4Q(Low)	134(85.1)	24(14.9)	
Educational status			
≤ Primary school	219(92.9)	20(7.1)	< 0.001
Middle school	161(92.8)	12(7.2)	
High school	506(87.9)	70(12.1)	
≥ College	519(82.6)	105(17.4)	
Marital status			
No	303(78.9)	83(21.1)	< 0.001
Yes	1,102(90.4)	124(9.6)	
Problem drinking			
No	1,329(87.8)	183(12.2)	0.001
Yes	76(74.9)	24(25.1)	
Smoking			
Non-smoking	1,260(87.8)	172(12.2)	0.057
Ex-smoker	53(79.6)	15(20.4)	
Current smoking	92(82.2)	20(17.8)	
Exercise			
No	1,219(87.7)	166(12.3)	0.036
Yes	186(81.7)	41(18.3)	
Sleep duration			
≤ 6 h	565(85.3)	92(14.7)	0.159
> 6 h	840(88.1)	115(11.9)	
Obesity			
Under weight	80(81.0)	21(19.0)	0.172
Normal	1,009(87.0)	143(13.0)	
Over weight	316(88.5)	43(11.5)	
Rheumatoid arthritis			
No	1,391(86.9)	206(13.1)	0.715
Yes	14(90.7)	1(9.3)	
Osteoarthritis			
No	1,296(86.9)	191(13.1)	1.000
Yes	109(86.9)	16(13.1)	
Stress			
Low	941(90.1)	108(9.9)	< 0.001
High	464(81.1)	99(18.9)	
Self-rated health			
Excellent/good	639(87.6)	94(12.4)	0.520
Fair/bad	766(86.4)	113(13.6)	
Depressive symptoms			
No	1,193(87.2)	172(12.8)	0.389
Yes	212(85.0)	35(15.0)	

^a^χ2 test

### The prevalence of TMD according to work-related factors and the risk of TMD by working hours

Non-manual workers were found to have a higher prevalence of TMD (15.6%) than manual workers (6.4%) (Table 2). Participants who worked less than 40 h had the lowest TMD prevalence (8.0%), followed by those working 40–48 h (12.6%), 49–60 h (15.2%), and more than 60 h (19.8%). Although working hours and occupation differ significantly in the prevalence of TMD, working schedule and employment status did not show any significant differences.

**Table 2:** The prevalence of temporomandibular disorders (TMD) according to work-related factors

	Prevalence of TMD		
	No N(%)	Yes N(%)	P value^a^
Total	1,405	207	
Occupation			
Manual	445(93.6)	32(6.4)	< 0.001
Non-manual	960(84.4)	175(15.6)	
Working schedule			
Daytime	1,208(86.7)	180(13.3)	0.600
Shift work	197(88.2)	27(11.9)	
Employment status			
Regular	1,009(86.2)	155(13.8)	0.198
Temporary	396(88.8)	52(11.2)	
Working hours (hrs/wk)			
< 40	244(92.0)	25(8.0)	0.011
40–48	752(87.4)	107(12.6)	
49–60	283(84.8)	48(15.2)	
> 60	126(80.2)	27(19.8)	

^a^χ2 test

We found no association between occupation and TMD (OR 1.69, 95% CI 0.99–2.88) in model 1. After adjusting for working schedule, employment status, and working hours, however, we found the association between occupation and TMD (OR 1.72, 95% CI 1.01–2.91). We found no association between shift work and TMD (OR 0.84, 95% CI 0.51–1.39). In addition, no association was found between temporary work and TMD (OR 1.14, 95% CI 0.75–1.72). In the case of working hours, based on 40 h less per week, ORs for women who worked 40–48 h, 49–60 h, and more than 60 h were 1.65 (95% CI 1.02–2.67), 2.05 (95% CI 1.17–3.60), and 2.82 (95% CI 1.49–5.35), respectively (Table 3). After adjusting for general characteristics including age, educational level, marital status, problem drinking, exercise, and stress, ORs for women who worked 40–48 h, 49–60 h, and more than 60 h per week were 1.16 (95% CI 0.69–1.93), 1.39 (95% CI 0.78–2.46), and 2.41 (95% CI 1.28–4.53), respectively (Model 1). In model 2, we adjusted for working schedule, employment status, and occupation as well as the variables in model 1. ORs for respondents who worked 40–48 h, 49–60 h, and more than 60 h were 1.16 (95% CI 0.69–1.94), 1.41 (95% CI 0.79–2.54), and 2.43 (95% CI 1.29–4.59), respectively. We found no association between shift work and TMD (OR 0.84, 95% CI 0.51–1.39). In addition, no association was found between temporary work and TMD (OR 1.14, 95% CI 0.75–1.72).

**Table 3:** The risk of TMD according to work-related factors

	Unadjusted		Model 1^a^		Model 2^b^	
	OR	95% CI	OR	95% CI	OR	95% CI
Occupation						
Manual			1		1	
Non-manual			1.69	0.99–2.88	1.72	1.01–2.91
Working schedule						
Daytime			1		1	
Shift work			0.85	0.51–1.40	0.84	0.51–1.39
Employment status						
Regular			1		1	
Temporary			1.05	0.70–1.57	1.14	0.75–1.72
Working hours (hrs/wk)						
< 40	1		1		1	
40–48	1.65	1.02–2.67	1.16	0.69–1.93	1.16	0.69–1.94
49–60	2.05	1.17–3.60	1.39	0.78–2.46	1.41	0.79–2.54
> 60	2.82	1.49–5.35	2.41	1.28–4.53	2.43	1.29–4.59

^a^adjusted for general characteristics (i.e., age, educational status, marital status, problem drinking, exercise, and stress)

^b^adjusted for general characteristics (i.e., age, educational status, marital status, problem drinking, exercise, and stress) and other work-related factors

## Discussion

This cross-sectional study investigated the association between work-related factors and TMD. Among work-related factors, the association between non-manual occupation and TMD and the association between working hours and TMD was significant. This study found that the risk of TMD was higher among women who worked more than 60 h per week than among those who worked less than 40 h per week, after adjusting for the general characteristics and work-related factors of this study population. However, the association between TMD and other work-related factors such as shift work and temporary work was not significant.

Younger women were more likely to have a higher TMD prevalence rate (Table 1). This result is consistent with the findings of other study [9]. Young women have a lower threshold for pain and they are more likely to perceive TMD symptoms than men [11]. Women that has a higher educational level showed a higher TMD prevalence rate (Table 1). A study showed jaw dysfunction symptoms were associated with higher education level [20]. Significant associations of pain in the orofacial region have been found with both higher and lower educational levels [21]. The association between educational level and TMD is not conclusive. About marital status in this study, single female workers showed a higher TMD prevalence rate (Table 1). A study reported that unmarried status correlated with poorer health [22]. Another study, however, suggested no correlation between marital status and TMD and whether a correlation exists between them is controversial [23]. For drinking, problem drinkers’ TMD prevalence rate was higher (Table 1). Miettinen O et al. reported that drinking at least once a week correlated with TMD symptoms [24]. The hypothalamic–pituitary–adrenal (HPA) axis’s dysregulation observed in chronic active alcoholism is closely related to psychiatric stress-related disorders, and TMD patients had higher prevalence rates for these disorders [25–27].

According to a 2015–2016 OECD report, the annual number of working hours in Korea was 2,464 in 2002 and this has declined each year. Nonetheless, the annual number of working hours in 2014 stood at 2,124, the second-highest among the OECD member states next to Mexico [28]. The number of working hours per week is limited to no more than 48, including overtime hours, according to the EU [16]. In Korea, death by overwork is considered for those working 60 h or more per week [17].

There are few researches to directly show the association between long working hours and TMD. However, many studies revealed a correlation between long working hours and psychiatric and musculoskeletal problems [29, 30]. Meanwhile, biologic (joint trauma), behavioral (psychiatric problems), environmental (head and cervical posture), and cognitive factors (pain threshold) all play a role in the development of TMD symptoms [31]. Since TMD is a disorder that presents with symptoms of the temporomandibular joint and masticatory muscles, it can also be considered a musculoskeletal disorder. Accordingly, we assume that mental and physical stress arising from long working hours affected TMD prevalence.

Chen Y et al. reported that the job stress of female workers who worked for 48 h or more per week was 1.79 times as that of women who worked for fewer than 48 h [32]. In the prospective Whitehall II cohort study, showed an excess risk of depression (hazard ratios [HR] 2.67, 95% CI 1.07–6.68) and anxiety (HR 2.84, 95% CI 1.27–6.34) associated with long working hours among women [33].

And many studies have supported that psychosocial factors related to long working hours have a correlation with TMD [13, 14]. In a case-control study, patients with TMD pain showed a higher level of anxiety (OR 5.1), somatization (OR 2.7), and depression (OR 3.5) than the control group [34]. Depression (incidence density ratio [IDR] 3.2), perceived stress (IDR 2.6), and mood (IDR 7.3) increased the risk of TMD [35]. A prospective cohort study on orofacial pain confirmed several psychological variables (stress, previous stressful life events, and negative affect) that could predict the onset of TMD pain [36].

In this study results, the TMD prevalence rate was significantly high for workers who perceived stress a lot (Table 1). Although we might think of the role of stress in the relationship between long working hours and TMD, the stress used in this study was not evaluated using a quantitative stress assessment tool but using only a questionnaire survey on stress perception in general. Therefore, it is not enough to regard the stress of our research as stress associated with long working hours. A more detailed analysis would be needed in the future, using an assessment tool that can accurately show a correlation between stress and long working hours.

Although the participant group with depressive symptoms showed a higher prevalence of TMD than the group with no depressive symptoms, the difference was not significant (Table 1). One possible reason of inconsistent findings with other previous studies could be that we used a single question to evaluate whether someone had depression; therefore this could affect the validity of our results. This should be addressed in future follow-up research.

In this study, we found no significant differences between day work and shift work when it came to prevalence of TMD. One of the reasons could be that there was possible underestimation owing to the healthy worker effect. Another reason is too small number of study subjects in this study.

In addition, we assume that satisfaction with shift work, not shift work itself, is associated with TMD. Symptoms related to TMD were correlated with alexithymia and depressive mood [37]. Dissatisfaction with shift work, not shift work itself, has been reported to have a positive correlation with depressive mood [37]. Kim et al. reported that whereas shift work increased metabolism risk factors for cardiovascular diseases or had a negative effect on mental health, shift work offers less responsibility outside of normal working hours and greater economic reward than day work [38]. Accordingly, it is necessary to more accurately evaluate the level of job satisfaction arising from shift work.

When the association between type of employment and TMD was examined, the TMD risk among temporary workers (OR 1.14, 95% CI 0.75–1.72) was higher but it was not significant (Table 3). According to 2016 data of Statistics Korea [39], 40.3% of female wage earners in Korea are temporary workers; the percentage of temporary female workers (27.8%) was lower in our research. It is believed that different definitions and assessment methods of temporary workers are used, which would result in different percentages of temporary workers and ultimately different TMD prevalence rates among published studies. Accordingly, future research should be conducted using an agreed definition, if possible, rather than a questionnaire as in our study, to identify whether a participant is a temporary worker.

Only few people have difficulty in daily life owing to TMD. However, such disorders worsen while unnoticed, and patients miss the right treatment timing and the disorders develop into chronic ones. Greene et al. reported that chronic TMD pain can result in the absence or loss of work or social interaction, and ultimately reduces the overall quality of life [40]. In the United States, it is estimated that 17.8 million working days are lost annually for 100 million full-time adult workers due to serious TMD [41].

The following limitations should be noted in this study. First, this research was a cross-sectional study to identify the association between work-related factors and TMD, therefore, we were unable to find any causal relationships. Second, this study was unable to reflect the double burden of female full-time workers. Third, information error may have been introduced during data collection using the questionnaire. We only checked whether participants worked shifts and did not evaluate their levels of satisfaction or stress regarding shift work, which would have resulted in a more compelling conclusion. In addition, in this study, participants were evaluated using only the questionnaire, without establishing a clear definition of regular and temporary workers. Lastly, various confounding variables, such as oral and maxillofacial habits that could affect TMD (clenching and bruxism), were not considered in this research. Tooth contacting habits (TCH) such as clenching and bruxism are considered to have an effect on the incidence, continuation, and deteriorating condition of TMD [42]. However, we did not include evaluation items for clenching and bruxism owing to the nature of the data used, which could not accurately reflect these factors and therefore could not produce significant outcomes. Follow-up research should incorporate evaluation items for clenching and bruxism to delve further into these factors.

Despite these limitations, our study is the first to address the associations between work-related factors and TMD using representative national data in Korea. There are various factors that contribute to TMD. If TMD is treated at an appropriate time and by considering various factors, the quality of life for people with TMD would be greatly improved. In addition, we believe it can reduce socioeconomic loss resulting from TMD.

## Conclusions

In this study, we found the risk of TMD was higher among women with long working hours. We believe that this study holds significance, as it provides basic data on the negative effects of long working hours on health.

## Abbreviations

AAOP: The American Academy of Orofacial Pain
BMI: Body mass index
CI: Confidence interval
EU: European Union
HR: Hazard ratio
IDR: Incidence density ratio
KNHANES: Korea National Health and Nutrition Examination Survey
OR: Odds ratio
RDC/TMD: Research diagnostic criteria for temporomandibular disorders
TCH: Tooth contacting habits
TMD: Temporomandibular disorders
